# Short- and Long-Term Outcomes of Indocyanine Green Fluorescence Navigation- Versus Conventional-Laparoscopic Hepatectomy for Hepatocellular Carcinoma: A Propensity Score-Matched, Retrospective, Cohort Study

**DOI:** 10.1245/s10434-022-13027-5

**Published:** 2023-01-16

**Authors:** Fusheng Liu, Haitao Wang, Weijie Ma, Jinghua Li, Yingyi Liu, Shengli Tang, Kun Li, Ping Jiang, Zhiyong Yang, Yueming He, Zhisu Liu, Zhonglin Zhang, Yufeng Yuan

**Affiliations:** 1 Department of Hepatobiliary and Pancreatic Surgery, Zhongnan Hospital of Wuhan University, Wuhan, Hubei People’s Republic of China; 2 Clinical Medicine Research Center for Minimally Invasive Procedure of Hepatobiliary & Pancreatic Diseases of Hubei Province, Wuhan, Hubei People’s Republic of China; 3 TaiKang Center for Life and Medical Sciences, Wuhan University, Wuhan, Hubei People’s Republic of China

## Abstract

**Background:**

Indocyanine green (ICG) fluorescence imaging technology is increasingly widely used in laparoscopic hepatectomy. However, whether it can provide long-term survival benefits to patients with liver malignancies remains unclear. This study investigated the clinical effect of laparoscopic hepatectomy for hepatocellular carcinoma (HCC) using ICG imaging technology.

**Methods:**

We retrospectively analyzed HCC patients who underwent laparoscopic hepatectomy at Zhongnan Hospital of Wuhan University from January 2016 to December 2020. Propensity score matching (PSM) was used to match patients undergoing ICG fluorescence navigation laparoscopic hepatectomy (ICG-FNLH) with those undergoing conventional laparoscopic hepatectomy (CLH) in a 1:1 ratio to minimize the influence of confounding factors. We compared perioperative status and long-term prognosis between the two groups and performed multivariate analysis to identify risk factors associated with overall survival and recurrence-free survival.

**Results:**

The original cohort consisted of 141 patients, with 50 patients in each group (100 patients in total) after PSM. The anatomical liver resection rate, R0 resection rate, and resection margin distance in the ICG-FNLH group were higher than those in the CLH group. The intraoperative blood loss was lower than that in the CLH group. The recurrence-free survival and overall survival of the ICG-FNLH group were better than those of the CLH group. ICG-FNLH improved the recurrence-free survival of HCC patients (hazard ratio [HR] = 2.165, 95% confidence interval [CI]: 1.136-4.127, *P* = 0.024).

**Conclusions:**

Compared with CLH, ICG-FNLH can improve the recurrence-free survival rate of patients with hepatocellular carcinoma and may help to improve the long-term prognosis of patients.

Primary liver cancer (PLC) is the third-leading cause of cancer-related death worldwide, and China accounts for more than half of the global morbidity and mortality.^1,2^ Hepatocellular carcinoma (HCC) is the most common type of PLC, accounting for approximately 90% of all cases.^3–5^ Hepatectomy, as an essential treatment for hepatocellular carcinoma, has made continuous progress in the past few decades, significantly improving the safety and efficacy of surgery. However, more than 70% of patients relapse within 5 years after surgery.^5,6^ To reduce the postoperative recurrence rate, factors, such as the detection of micrometastases, R0 resection, and wide resection margins, have attracted increasing attention.^7–9^ Anatomical resection also is a crucial surgical technique, because it can avoid the problems of tissue necrosis, abscess, and liver failure caused by postoperative liver parenchyma ischemia. Several studies have shown that anatomical resection can reduce early recurrence and mortality in patients.^10,11^

Indocyanine green (ICG) is a fluorescent dye, approved by the U.S. Food and Drug Administration (FDA), that can rapidly bind to plasma proteins after intravenous injection and exhibits autofluorescence under specific wavelength illumination.^12^ In liver surgery, preoperative peripheral intravenous injection of ICG can achieve intraoperative identification of liver tumors. ICG in vivo is excreted by normal liver tissue, but tumor cells with different pathological types or degrees of differentiation have unequal metabolic capacities for ICG, manifesting as total fluorescence, partial fluorescence, or ring fluorescence.^13,14^ Based on this feature, ICG can guide tumor resection or identify invisible lesions. On the other hand, surgeons can use intraoperative ICG fluorescence navigation to mark the boundary of the target segment and achieve anatomical resection.^14^

Regarding ICG fluorescence-navigated hepatectomy, the initial literature focused on single-case or several-case reports to demonstrate the technical model and procedure,^15–17^ and several teams successively reported intraoperative and perioperative results.^18–22^ However, current studies mainly focus on describing surgical techniques and short-term postoperative outcomes. Pathological types of research cases include primary liver cancer, metastases, and benign liver tumors. Most studies have few cases, and some studies did not use established controls. Reports on the long-term effects of ICG-FNLH for hepatocellular carcinoma are even scarcer.^23^ To our knowledge, there are currently no studies reporting that ICG navigation hepatectomy improves long-term prognosis in patients with HCC. This study was designed to investigate whether there is a statistically significant difference in the outcome of ICG fluorescence imaging on recurrence-free survival (RFS) and overall survival (OS) in patients with hepatocellular carcinoma compared with conventional laparoscopic hepatectomy.

## Clinical Data and Methods

### Patient Selection Criteria

**Fig. 1:**
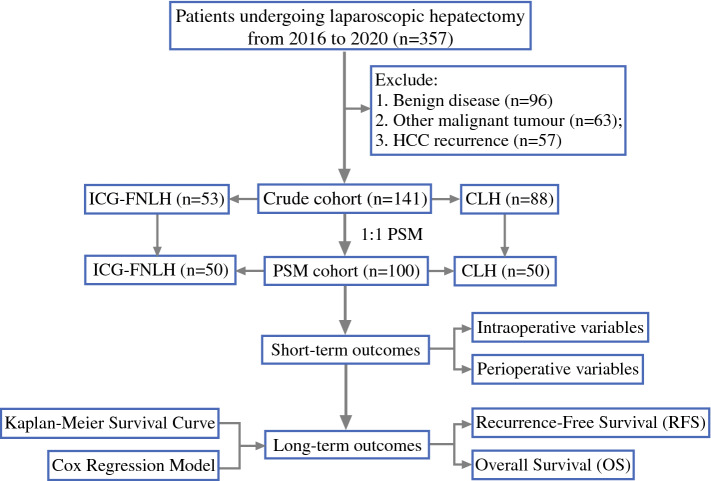
Enrollment and conduct process

From January 2016 to December 2020, a total of 357 patients underwent laparoscopic hepatectomy at the Department of Hepatobiliary Pancreatic Surgery in Zhongnan Hospital of Wuhan University. After filtering, 141 patients were included in this study and divided into the ICG-FNLH group and the CLH group (Fig. 1). The inclusion criteria were as follows: (1) histopathological diagnosis of hepatocellular carcinoma; (2) laparoscopic hepatectomy surgical approach, including anatomical or nonanatomical hepatectomy; and (3) in ICG-FNLH cases, the criteria for successful implementation were met: When used for tumor imaging, the ICG washes out well from the liver parenchyma, and the tumor fluorescence has good contrast with the liver; when used for fluorescence imaging of liver segments, the fluorescence imaging of positive or negative staining is satisfactory, and the boundary with the adjacent segment is clear, which can guide the operation of liver cutting. The following exclusion criteria were used: (1) patients with a pathological result other than hepatocellular carcinoma; (2) patients who underwent previous liver surgery for the tumor or other reasons; (3) patients who were unable to tolerate hepatectomy due to insufficient residual liver function; (4) patients with unsatisfactory fluorescence imaging (ICG-FNLH group); and (5) patients with severe heart, lung, kidney, or other organ dysfunction. This study complies with the Declaration of Helsinki and was approved by the Ethics Committee of Zhongnan Hospital of Wuhan University, authorization number: Kelun[2020100].

### Surgical Procedure

Experienced liver surgeons at the center performed the surgery. Surgical procedures included anatomical or nonanatomical hepatectomy based on tumor characteristics, size, location, and adjacent structures. The criteria for anatomical hepatectomy are technically defined as follows: marking the border of the liver segment on the liver surface by staining or ischemia line; ultrasound-guided liver parenchyma cutting with the landmark vein of the liver segment as the border; exposure of all veins of significance; and ligation of the Glisson system near the root of the liver segment.^24,25^

The imaging equipment was the Canadian Pinpoint Novadaq laparoscopic fluorescence imaging system, which uses LED visible light and near-infrared dual light sources and cooperates with the fluorescent contrast agent to display endoscopic images. The system provides three imaging modes: high-definition natural light view, black and white view, and green fluorescence view. The standard concentration of ICG for injection (25 mg/ampoule) was 2.5 mg/ml diluted with 10 ml of sterile water, provided by Weicai (Liaoning) Pharmaceutical Co., Ltd. The application methods of ICG fluorescence imaging included fluorescence imaging of liver tumors injected with ICG before surgery and positive or negative fluorescence staining of liver segments injected with ICG during surgery.

**Fig 2:**
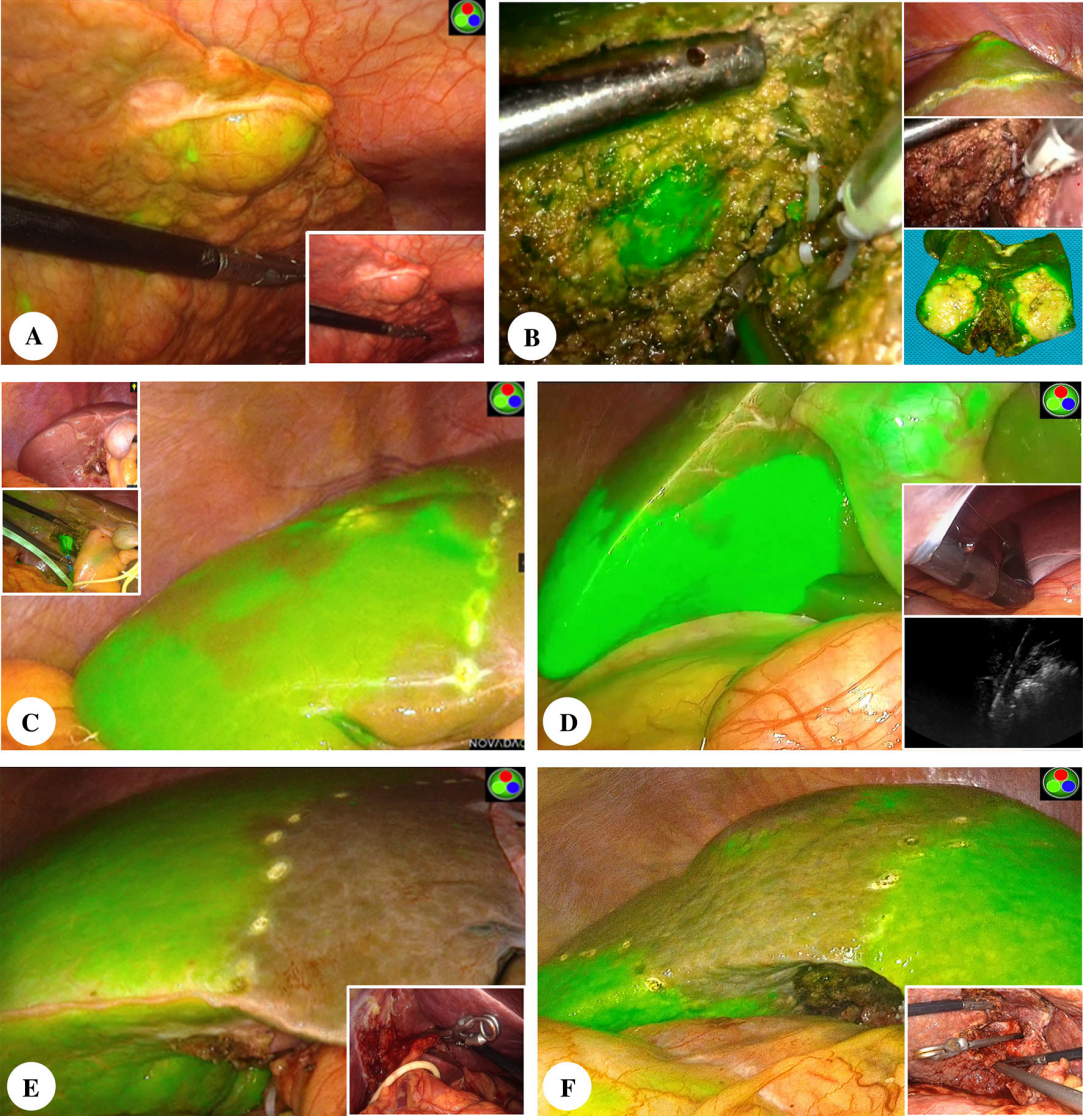
Use of ICG fluorescence-navigated laparoscopic hepatectomy. Fluorescence tumor imaging. **A** Fluorescence visualization helps to identify malignant nodules at the liver margins in severe cirrhosis with postoperative pathology showing hepatocellular carcinoma. **B** Mark the tumor boundary by fluorescence imaging during the operation; the right panel shows the liver surface visual field, conventional visual field, and the fluorescence performance of postoperative specimens. Positive fluorescent staining: **C** Laparoscopic transhepatic puncture (S6+part of S5), the left panel shows the portal vein branch of the target liver segment being dissected under direct vision and injected with indocyanine green (ICG); **D** Laparoscopic transhepatic puncture (S6), the right panel shows the intraoperative ultrasound-guided puncture of the portal vein branch of the target hepatic segment followed by injection of ICG; Negative fluorescent staining: **E** Fluorescence imaging of peripherally injected ICG after clamping of the left hepatic Glissonean pedicle; **F** Fluorescence imaging of peripheral intravenous injection of ICG after clamping the Glissonean pedicle of the right anterior lobe (S5+S8)

For tumor imaging, ICG was injected 2–4 days before surgery, and the standard dose was 0.5 mg/kg; patients with apparent liver cirrhosis were injected 4-6 days before surgery. When used for liver segment imaging, 1 ml (2.5 mg) of the above standard concentration of ICG solution was diluted with normal saline to 100 ml (or 2.5 ml diluted to 250 ml), and the dose was adjusted according to the target liver segment volume. For positive staining of the liver segment, 1–10 ml of the above-diluted solution was injected through the portal vein of the target liver segment. For negative staining, 10–20 ml of the above solution was injected through the peripheral vein after clipping the blood supply vessel of the target liver segment, and the injection volume was appropriately increased if the fluorescence was not satisfactory. Figure 2 shows the primary method of laparoscopic hepatectomy using ICG fluorescence imaging at our center (Fig. 2).

### Data Collection and Follow-up

All patient data were obtained from the center's clinical database. Demographics, perioperative clinical data, and follow-up outcomes were collected. Preoperative data included sex, age at surgery, body mass index (BMI), American Society of Anesthesiologists (ASA) score, routine blood test results, liver and kidney function, tumor markers, infectious disease examination, and abdominal contrast-enhanced CT or MRI. Intraoperative indicators included operative procedure, operative time, blocking times and duration of hepatic blood flow, intraoperative blood loss, and blood transfusion. Pathological data included tumor number, degree of differentiation, and margin distance. Postoperative information included routine blood test results; liver and kidney function on the first, third, and seventh postoperative days; length of postoperative hospital stay; and postoperative complications. Follow-up data were recurrence-free survival and overall survival.

According to the preoperative hematological examination, radiological examination, and postoperative pathological report, the patients were evaluated for tumor staging according to different standards, including Okuda stage,^26^ TNM 8^th^ edition (according to the American Joint Committee on Cancer) stage,^27^ Barcelona Liver Cancer (BCLC) stage,^28^ Chinese Liver Cancer (CNLC) stage,^29^ Cancer of the Liver Italian Program (CLIP) score and Japan Integrated Staging (JIS) score.^30,31^ Major hepatectomy is defined as the extension of resection of ≥3 liver segments according to the Couinaud classification.^32^ The positive margin (R1) was defined as the presence of tumor cells in the transverse section under the microscope. The lowest values of platelets (PLT) and albumin (ALB) and the highest values of total bilirubin (TBIL) and alanine aminotransferase (ALT) were recorded from the examination results 1, 3, and 7 days after surgery. Postoperative complications were graded according to the Clavien–Dindo system.^33^

Telephone follow-ups were conducted every 3 months after surgery. In principle, patients with high recurrence risk underwent ultrasound and AFP examinations every 2 months for 2 years after surgery and enhanced CT or MRI, chest x-ray, or CT scans every 3 months. After 2 years, the frequency of ultrasound and AFP was extended to every 3 months, and the frequency of CT or MRI was extended to every 6 months. The time of relapse discovery and death were recorded in detail. If patients were lost to follow-up before relapse or death, the time of the last follow-up was recorded.

The main observation results of this study were recurrence-free survival (RFS) and overall survival (OS) of the two groups of patients, and secondary observations included surgical margins, intraoperative blood loss, and postoperative complications.

### Statistical Analysis

A propensity score-matching model was used to minimize selection bias due to insufficient randomization, the matching tolerance was set to 0.2, and the ratio was set as 1:1. The propensity score was calculated based on the following parameters: sex, age, BMI, ASA grade, PLT, ALT, TBIL, ALB, cirrhosis, HBV infection, alpha-fetoprotein (AFP) grade, tumor number, maximum tumor diameter, degree of differentiation, Child–Pugh grade, Okuda stage, TNM stage, BCLC stage, CNLC stage, CLIP score, and JIS score.

Qualitative variables are expressed as numbers or percentages, and quantitative variables are expressed as the mean ± standard deviation or median (range) depending on the normality of the distribution. Pearson’s chi-square test or Fisher’s exact test was used to compare qualitative variables, and the independent sample *t* test or Mann–Whitney *U* test was used to compare quantitative variables. Survival analysis was performed using recurrence-free survival and overall survival, the Kaplan–Meier method to calculate survival curves, and the log-rank test for survival comparisons. Important prognostic factors affecting long-term survival were analyzed using a Cox proportional hazards regression model, and the results of the Cox regression analysis were expressed as hazard ratios (HR) and corresponding 95% confidence intervals (95% CIs). *P* value < 0.05 was considered statistically significant. Statistical analysis was performed using the SPSS software package (version 26).

## Results

### Baseline Features and PSM

A total of 141 patients with hepatocellular carcinoma were included in the original cohort, including 53 patients in the ICG-FNLH group and 88 patients in the CLH group (Fig. 1). Before PSM, the two groups’ maximum tumor diameter (35 mm vs. 50 mm, *P* = 0.030) was significantly different. After PSM, 50 patients were included in each cohort, and there was no significant difference in baseline characteristics (Table 1).

**Table 1:** Comparison of baseline conditions before and after propensity score matching (PSM)

Items	Total *N* = 141	Before PSM			After PSM		
		ICG-FNLH *n* = 53 (%)	CLH *n* = 88 (%)	*P* value	ICG-FNLH *n* = 50 (%)	CLH *n* = 50 (%)	*P* value
Gender, male (%)	123 (87.23)	49 (92.45)	74 (84.09)	0.150^a^	46 (92)	42 (84)	0.218^a^
Age	57.62 ± 10.32	57.26 ± 10.27	57.84 ± 10.4	0.749^b^	56.82 ± 10.41	59.16 ± 10.82	0.273^b^
BMI	22.98 ± 3.46	22.91 ± 3.37	23.02 ± 3.53	0.846^b^	22.94 ± 3.27	23.55 ± 3.7	0.383^b^
ASA grade (I/II/III)	10/98/33	1/41/11	9/57/22	0.764^c^	1/39/10	3/30/17	0.261^c^
PLT	149.0 (115.5,192.0)	134 (112,181)	158.58 ± 56.01	0.212^c^	134 (112.5,184.5)	154.24 ± 54.39	0.682^c^
ALT	26.0 (19.0,38.0)	32 (21,42.5)	25 (18.25,35.75)	0.064^c^	31 (20.75,41.25)	27 (19.75,36)	0.666^c^
TBIL	14.7 (11.0,19.8)	16.05 ± 7.09	14.7 (11.5,19.68)	0.784^c^	16.25 ± 7.15	15.84 ± 6.21	0.753^b^
ALB	39.7 (36.8,42.4)	39.49 ± 3.67	40.2 (36.75,42.6)	0.318^c^	39.57 ± 3.67	39.8 (36.3,41.93)	0.920^c^
Cirrhosis (%)	76 (53.90)	31 (58.49)	45 (51.14)	0.396^a^	28 (56)	22 (44)	0.230^a^
HBV infection (%)	97 (68.79)	34 (64.15)	63 (71.59)	0.356^a^	33 (66)	34 (68)	0.832^a^
AFP (ng/ml)				0.273^c^			0.525^c^
≤20	69 (48.94)	28 (52.83)	41 (46.59)		27 (54)	24 (48)	
20~400	39 (27.66)	16 (30.19)	23 (26.14)		15 (30)	15 (30)	
400~1200	10 (7.09)	3 (5.66)	7 (7.95)		2 (4)	6 (12)	
≥1,200	23 (16.31)	6 (11.32)	17 (19.32)		6 (12)	5 (10)	
Multiple tumors (*n* > 1) (%)	33 (23.40)	13 (24.53)	20 (22.73)	0.807^a^	11 (22)	7 (14)	0.298^a^
Max tumor diameter(mm)	44.0 (30.0, 60.0)	35 (25,52.5)	50 (30,70)	**0.030**^**c**^	39 (25,55)	45 (30,60)	0.206^c^
Differentiation (I/II/III)	15/97/29	6/40/7	9/57/22	0.160^c^	6/37/7	5/33/12	0.248^c^
Child-Pugh grade (A/B/C)	137/4/0	51/2/0	86/2/0	0.603^a^	49/1/0	48/2/0	0.558^a^
Okuda stage (I/II/III)	121/20/0	48/5/0	73/15/0	0.210^a^	45/5/0	41/9/0	0.249^a^
TNM stage (I/II/III/IV)	62/54/21/4	27/19/6/1	35/35/15/3	0.153^c^	26/17/6/1	20/20/8/2	0.212^c^
BCLC stage (0/A/B/C)	11/78/47/5	4/29/18/2	7/49/29/3	0.858^c^	4/27/17/2	3/30/14/3	0.882^c^
CNLC grade (I/II/III/IV)	80/15/45/1	29/7/17/0	51/8/28/1	0.415^c^	28/5/17/0	30/3/16/1	0.406^c^
CLIP score <2 (%)	116 (82.27)	47(88.68)	69 (78.41)	0.578^c^	45 (90)	44 (88)	0.659^c^
JIS score <2 (%)	64 (45.39)	22 (41.51)	42 (47.73)	0.850^c^	22 (44)	25 (50)	0.991^c^

Bold value indicates the statistically significant result

^a^Chi-square test; ^b^Independent samples *t* test; ^c^Mann-Whitney *U* test

### Surgical Conditions of ICG Fluorescence Navigation

Patients undergoing hepatectomy using ICG fluorescence imaging were injected with ICG preoperatively to prepare for intraoperative tumor visualization. The inclusion criteria of ICG-FNLH included satisfactory fluorescence imaging and a clear boundary with the adjacent liver tissue, which guided the hepatectomy operation. Among the 50 patients, only 27 patients underwent liver tumor fluorescence imaging, and the preoperative injection time of ICG was 2–4 days. Eight cases successfully used positive fluorescent staining of the liver segment, and the dose of ICG injected directly or under ultrasound guidance through the portal vein branch was generally 5–10 ml (0.125–0.25 mg). Negative staining was successfully used in 15 cases. Glisson’s pedicle of the target liver segment was ligated during the operation, and ICG was injected peripherally at a dose of 10–20 ml (0.25–0.5 mg). Figure 2 shows the intraoperative application of ICG fluorescence navigation (Fig. 2).

### Intraoperative Comparison

After matching, the ICG-FNLH group performed significantly better than the CLH group in terms of surgical indicators (Table 2). The ICG-FNLH group had a higher anatomic hepatectomy rate (66% vs. 42%, *P* = 0.016), R0 resection rate (96% vs. 82%, *P* = 0.025), and resection margin distance (1.30 cm vs. 0.10 cm, *P* = 0.030) than those in the CLH group. The conversion rate to laparotomy (2% vs. 14%, *P* = 0.025) and intraoperative blood loss (700 vs. 1083.33 ml, *P* = 0.004) were lower than those in the CLH group. The above differences were all statistically significant. The operation time and the number and duration of blood flow occlusions in the ICG-FNLH group were higher than those in the CLH group, but the differences were not statistically significant.

**Table 2:** Comparison of short-term outcomes

Items	ICG-FNLH, *n* = 50	CLH, *n* = 50	*P* value
*Intraoperative situation*			
Anatomical hepatectomy, *n* (%)	33 (66)	21 (42)	**0.016**^**a**^
Major hepatectomy, *n* (%)	7 (14)	13 (26)	0.086^a^
Conversion to laparotomy, *n* (%)	1 (2)	7 (14)	**0.025**^**a**^
Operation time, min	412.8 ± 116.21	402.33 ± 84.49	0.221^b^
Blood flow occlusion	48 (96)	34 (68)	**0.002**^**a**^
Number	4.4 ± 2.3	3.5 ± 2.59	0.315^b^
Duration	76.6 ± 43.4	55.33 ± 42.46	0.092^b^
Blood loss, ml	700 ± 200	1083.33 ± 240.14	**0.004**^**b**^
Blood transfusion, *n* (%)	11 (22)	17 (34)	0.181^a^
Plasma, ml	400 (375,400)	350.0 (262.5,650.0)	0.383^c^
Red blood cells, u	2.00 (2.00,2.50)	2.50 (2.00,3.25)	0.058^c^
R0 resection, *n* (%)	48 (96)	41 (82)	**0.025**^**a**^
Margin distance (cm)	1.30 (0.45,2.00)	0.10 (0.08,1.20)	**0.030**^**c**^
*Perioperative situation*			
Postoperative hospital stay	10 (7,12)	9 (7,13)	0.978^c^
PLT	101 (90,142.5)	106 (86,151)	0.845^c^
INR	1.26 ± 0.16	1.16 ± 0.14	**0.003**^**b**^
TBIL	23.8 (21.1,32.2)	24.3 (20.05,37.3)	0.908^c^
ALT	541 (218,820)	208 (116.5,346.5)	**0.022**^**c**^
ALB	32.6 ± 3.64	30.96 ± 4.66	0.732^b^
CREA	75.95 ± 16.1	77.2 ± 21.27	0.558^b^
Complications, *n* (%)	38	38	1.000^a^
Bile leakage	6	6	1.000^a^
Abdominal bleeding	0	1	0.315^a^
Ascites	14	14	1.000^a^
Pleural effusion	23	17	0.221^a^
Atelectasis	14	8	0.148^a^
Intestinal dysfunction	8	7	0.779^a^
Subcutaneous emphysema	3	2	0.646^a^
Abdominal infection	0	5	**0.022**^**a**^
Chest infection	3	5	0.461^a^
Clavien-Dindo grade			0.886^c^
I	5	5	
II	22	21	
III	11	12	

Bold values indicate the statistically significant result

^a^Chi-square test; ^b^Independent samples *t* test; ^c^Mann-Whitney *U* test

### Short-Term Postoperative Outcomes

The highest values of INR (1.26 vs. 1.16, *P* = 0.003) and ALT (541 vs. 208 U, *P* = 0.022) in the ICG-FNLH group were higher than those in the CLH group within 1 week after surgery, and the incidence of postoperative abdominal infection in the ICG-FNLH group was lower than that in the CLH group (0 vs. 10%, *P* = 0.022); the difference was statistically significant. Other short-term postoperative outcomes, such as postoperative hospital stay and postoperative complications, were not significantly different (Table 2).

### Long-Term Postoperative Outcomes

After PSM, the mean follow-up time for surviving patients was 34.15 ± 16.43 months in the entire cohort, 26.99 ± 10.76 months in the ICG-FNLH group, and 41.32 ± 18.02 months in the CLH group. Thirty-seven (37%) of 100 patients relapsed, and 22 (22%) died. The 6-month and 18-month recurrence-free survival rates of the ICG-FNLH group were 90% and 80%, respectively, and the overall survival rates were 98% and 88%, respectively. The 6-month and 18-month, recurrence-free, survival rates in the CLH group were 82% and 66%, respectively, and the overall survival rates were 98% and 84%, respectively. In addition, the 3-year recurrence-free survival and overall survival rates in the CLH group were 48% and 68%, respectively. Because the follow-up time of some patients in the ICG-FNLH group was less than 3 years, the 3-year recurrence rate and survival rate were not calculated.

**Fig. 3:**
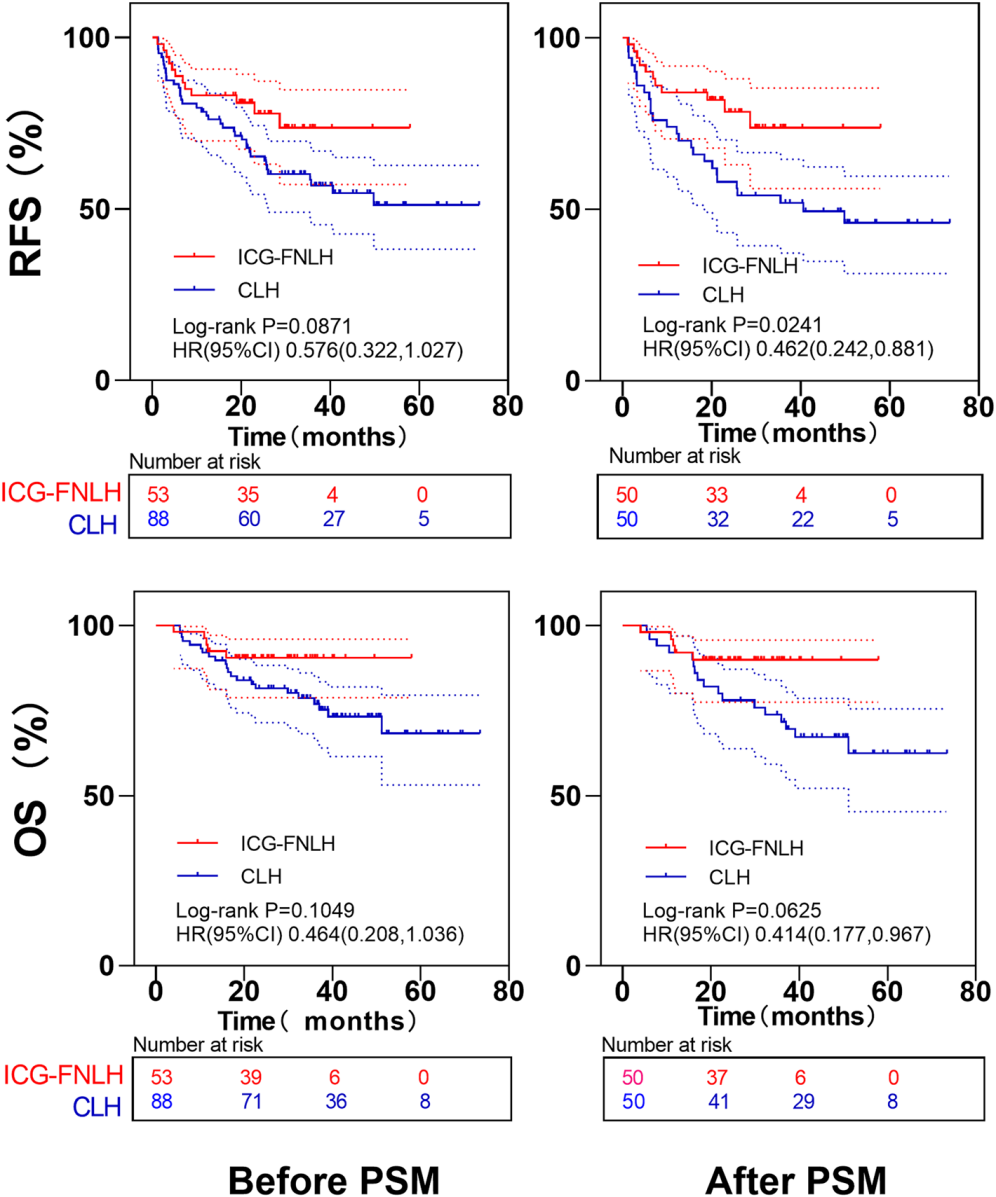
Comparison of recurrence-free survival rates and overall survival rates between the two groups before and after PSM. *ICG-FNLH* ICG fluorescence navigation laparoscopic hepatectomy; *CLH* conventional laparoscopic hepatectomy

Compared with the CLH group, the ICG-FNLH group had a prolonged recurrence-free survival (HR = 0.462, 95% CI: 0.242~0.881, log-rank *P* = 0.024), and the difference was statistically significant (Fig. 3). The overall survival time also was better than that of the CLH group (HR = 0.414, 95% CI: 0.177~0.967, log-rank *P* = 0.063), but the difference was not statistically significant (Fig. 3).

### Influencing Factors of RFS and OS

We performed Cox regression analysis on baseline characteristics and intraoperative outcomes predicting RFS and OS in the PSM cohort (Table 3). For RFS, univariate analysis showed that laparoscopic mode (HR = 0.449, 95% CI: 0.22~0.917, *P* = 0.028), presence of HBV infection, multiple tumors, degree of differentiation, TNM stage, BCLC stage, CNLC stage, CLIP score, JIS score, positive margin, and margin distance were associated with disease recurrence. Multivariate analysis showed that the presence or absence of HBV infection (HR = 2.937, 95% CI: 1.168~7.385, *P* = 0.022), degree of differentiation (HR = 2.686, 95% CI: 1.221~5.905, *P* = 0.014), and positive margin (HR = 6.858, 95% CI: 1.970~23.881, *P* = 0.002) were independent factors of tumor recurrence.

**Table 3:** Cox regression analysis of recurrence-free survival and overall survival

Items	Recurrence-free survival				Overall survival			
	Univariate analysis		Multivariate analysis		Univariate analysis		Multivariate analysis	
	HR (95% CI)	*P* value	HR (95% CI)	*P* value	HR (95% CI)	*P* value	HR (95% CI)	*P* value
Laparoscopic mode (ICG-FNLH)	0.449 (0.220,0.917)	**0.028**	0.465 (0.204,1.062)	0.069	0.392 (0.142,1.085)	0.072		
Gender (male)	0.880 (0.343,2.259)	0.79			1.623 (0.379,6.957)	0.514		
Age	0.978 (0.951,1.006)	0.119			0.964 (0.929,1.001)	0.054		
BMI	0.991 (0.903,1.088)	0.856			1.019 (0.907,1.144)	0.757		
Cirrhosis	1.202 (0.629,2.296)	0.577			1.021 (0.442,2.359)	0.961		
HBV infection	3.051 (1.271,7.321)	**0.012**	2.937 (1.168,7.385)	**0.022**	5.796 (1.354,24.813)	**0.018**	6.119 (1.400, 26.743)	**0.016**
AFP	1.239 (0.919,1.670)	0.161			1.281 (0.877,1.871)	0.2		
Multiple tumors	2.820 (1.333,5.965)	**0.007**	2.026 (0.657,6.249)	0.219	2.626 (1.003,6.872)	**0.049**	1.358 (0.317, 5.822)	0.68
Max tumor diameter	1.003 (0.990,1.015)	0.654			1.005 (0.989,1.021)	0.573		
Differentiation	3.103 (1.763,5.464)	**<0.001**	2.686 (1.221,5.905)	**0.014**	3.784 (1.916,7.472)	**<0.001**	3.241 (1.405, 7.473)	**0.006**
Okuda stage	1.740 (0.790,3.831)	0.169			1.823 (0.666,4.989)	0.242		
TNM stage	1.730 (1.195,2.506)	**0.004**	0.671 (0.317,1.419)	0.296	2.063 (1.301,3.272)	**0.002**	0.928 (0.401, 2.147)	0.861
BCLC stage	1.794 (1.122,2.866)	**0.015**	1.460 (0.722,2.952)	0.292	1.745 (0.991,3.074)	0.054		
CNLC stage	1.263 (1.068,1.493)	**0.006**	1.015 (0.732,1.407)	0.93	1.352 (1.080,1.694)	**0.009**	1.084 (0.749, 1.569)	0.669
CLIP score	1.727 (1.203,2.479)	**0.003**	1.141 (0.615,2.116)	0.676	1.754 (1.081,2.845)	**0.023**	0.817 (0.35, 1.909)	0.641
JIS score	2.578 (1.690,3.933)	**<0.001**	1.481 (0.656,3.342)	0.344	3.197 (1.777,5.751)	**<0.001**	1.575 (0.546, 4.544)	0.4
Anatomical hepatectomy	0.877 (0.458,1.677)	0.691			0.515 (0.213,1.244)	0.14		
Positive margins	10.699 (4.83,23.698)	**<0.001**	6.858 (1.970,23.881)	**0.002**	7.079 (2.943,17.03)	**<0.001**	7.305 (2.088, 25.56)	**0.002**
Margin distance	0.574 (0.341,0.964)	**0.036**	0.958 (0.569,1.612)	0.871	0.461 (0.207,1.024)	0.057		

Bold values indicate the statistically significant result

For OS, univariate analysis showed that the presence or absence of HBV infection, multiple tumors, degree of differentiation, TNM stage, CNLC stage, CLIP score, JIS score, and positive resection margin were associated with OS. Multivariate analysis showed that the presence or absence of HBV infection (HR = 6.119, 95% CI: 1.400, 26.743, *P* = 0.016), degree of differentiation (HR = 3.241, 95% CI: 1.405, 7.473, *P* = 0.006), and positive margin (HR = 7.305, 95% CI: 2.088, 25.56, *P* = 0.002) were independent factors for overall survival.

## Discussion

ICG can rapidly bind to plasma proteins and lipoproteins in a physiological environment to form aggregated dye molecules. The plasma protein-ICG complex in the blood circulation does not extravasate through the intercellular space of normal vascular endothelial cell but is actively taken up in the liver cell membrane and excreted into the bile duct through the cell membrane on the bile side capillary. ICG entering the biliary tract will combine with bile components and be completely excreted from the body without participating in enterohepatic circulation.^34,35^ ICG was initially used in liver surgery to guide the assessment of liver function before hepatectomy, especially in patients with cirrhosis. The 15-minute ICG retention rate (ICG-R15) has important guiding significance in accurately evaluating liver reserve function.^36^

Laparoscopic surgery lacks intraoperative palpation. Conventional laparoscopic liver resection is mainly based on visual judgment when identifying tumors, and sometimes it is challenging to avoid miscut tumors or margins that are too small. The ICG fluorescence imaging system helps to identify liver subcapsular tumors during laparoscopic surgery.^13,14,37^ The liver tumor's placeholder effect or growth process will lead to an abnormal local bile excretion environment, so the local tumor can still maintain relative fluorescence when the background liver ICG is cleared; the local fluorescence that can guide the operation is the advantage of fluorescence laparoscopy.

Intraoperative fluorescence can detect suspicious lesions that are difficult to find with the naked eye, allowing the operator to redefine the surgical tangent. Conversely, in the process of liver cutting, deviation of the surgical tangent often occurs, it is challenging to detect the departure of the tangent in conventional laparoscopy. However, the tumor's fluorescence border helps the operator to confirm the tumor location. Therefore, it is more accessible to adjust the transection plane and complete tumor resection under fluorescence laparoscopy (Fig. 2A, B).

The role of ICG in identifying tumors also has limitations. Studies have shown that ICG fluorescence imaging only offers a significant diagnostic advantage for subcapsular and superficial liver tumors because of its limited penetration ability.^37,38^ The discovery of deep tumors needs to be further judged by combining near-infrared-II region fluorescence (NIR-II, 1000–1700 nm) imaging and intraoperative ultrasound. In addition, the false-positive rate of ICG for intraoperative diagnosis is higher, which is more evident in patients with liver cirrhosis.^39^ In this case, the preoperative ICG injection time should be appropriately extended.

ICG fluorescence imaging also is used to mark the boundary of the target liver segment in laparoscopic liver resection, and the main methods are positive and negative fluorescence staining. During positive fluorescence staining, we performed the corresponding portal vein puncture after dissecting the target hepatic pedicle during the operation or directly punctured the corresponding portal vein under the guidance of percutaneous ultrasound (Fig. 2C, D). The negative fluorescence staining method was based on the Glissonean pedicle transection method (Fig. 2E).^40^ Compared with positive fluorescence staining, the negative staining technique avoids portal vein puncture and is easier to perform during surgery. However, dissection of the Glissonean pedicle in complex areas (such as S6, S7, and S8) is still challenging, requiring the operator to have superb experience with laparoscopic technology. It is worth noting that the dose of ICG is not ideal within the allowable range of pharmacology. An excessive dose is likely to cause the boundary of the target liver segment to infiltrate. We tried to use ICG at a dose as low as 0.0025 mg/kg during negative fluorescence staining, which also could satisfy the required fluorescence effect for surgery (Fig. 2F). Overall, the ICG required for liver segment staining tends to be low dose, with a higher rate of fluorescent labeling and more effortless adjustment.

In 2017, our center introduced ICG fluorescence imaging technology, initially only used for a small number of patients. With the gradual increase in the frequency of use, fluorescence laparoscopy has been applied to most laparoscopic hepatectomy patients since 2019. To minimize the impact of tumor heterogeneity on the long-term survival of tumor patients, this study specifically selected HCC as a pathological type to compare the prognosis of liver cancer patients. Our study suggests that ICG-FNLH helps prolong RFS in patients with hepatocellular carcinoma and may contribute to OS, although this conclusion may require more case studies and longer follow-up periods to support it.

The intraoperative results showed the advantages of ICG-FNLH compared with CLH for hepatocellular carcinoma. First, the rate of anatomical liver resection was significantly improved, which is closely related to the technical development of ICG fluorescent labeling of liver segments. Second, the rate of conversion to laparotomy and intraoperative blood loss were controlled, making it easier for surgery to follow the established plan. More importantly, thanks to the excellent performance of ICG fluorescence for tumor visualization, the closest margin distance and R0 resection rate of pathological specimens were significantly improved. In addition, tiny lesions that were unexpectedly discovered under fluoroscopic imaging were excised, further enhancing the effectiveness of the operation. Previous studies have also shown that ICG fluorescence navigation can help reduce intraoperative bleeding and obtain wider surgical margins, which is consistent with our conclusions.^22^

In short-term postoperative outcomes, the difference between ICG-FNLH and CLH was insignificant. The peak value of ALT within one week after operation in the ICG-FNLH group was higher than that in the CLH group, which may be related to the longer blood flow blocking time that causes hepatocytes to release more liver enzymes. Overall, the short-term results of the two groups of patients were similar, suggesting that the safety of ICG-FNLH is relatively stable. Multiple studies, including our previous study, have shown that the ICG-FNLH technique can be performed safely.^18,21,41^ Some studies also have shown that it can reduce the incidence of postoperative complications.^7,19^

In this study, laparoscopic mode, hepatitis B virus infection, multiple tumors, tumor differentiation, TNM stage, BCLC stage, CNLC stage, CLIP score, JIS score, positive margins, and margin distance were all related to the recurrence of the disease. Hepatitis B virus infection, tumor differentiation, and positive resection margin were independent influencing factors of tumor recurrence, which is consistent with previous studies.^9,42–44^ Among the predictors of long-term prognosis in patients with hepatocellular carcinoma, HBV infection, HCV infection, alpha-fetoprotein, tumor number, maximum tumor diameter, tumor differentiation, and microvascular invasion are all critical influencing factors.^45–48^ The various tumor staging standards proposed by integrating the above indicators further stratify the prognosis of patients with hepatocytes so that surgeons can more intuitively judge the patient’s condition and make clinical decisions.^5,29^ In our data, no statistically significant benefit in overall survival was observed with ICG fluorescence, suggesting that overall survival in HCC is affected by multiple factors, including tumor nature, and requires a comprehensive and multidimensional treatment approach to conduct disease management.

In recent decades, the emergence of new surgical concepts and medical technologies has led to the continuous optimization of the long-term prognosis of cancer patients. Surgical concepts and techniques, such as Pringle’s maneuver, the low central venous pressure concept, and anatomical hepatectomy, make hepatectomy safer and more standardized. Several studies have shown that anatomical liver resection can significantly improve long-term prognosis in patients with hepatocellular carcinoma.^10,11,49^ In our results, anatomical liver resection was not significantly associated with tumor recurrence, possibly due to the small number of samples and different surgical techniques. From our results, the improved prognosis of patients with ICG fluorescence navigation may be more closely related to R0 resection and resection margin distance, and the discovery of unexpected lesions and complete resection also may be important reasons. Therefore, in formulating surgical plans for different patients, the importance of anatomical resection may need to be carefully considered and performed to ensure surgical safety and adequate liver function. In terms of medical technology, extensive sample data have confirmed that laparoscopic hepatectomy is safe and effective and is superior to laparotomy in reducing bleeding and hospitalization time.^50^ Overall, advances in surgical techniques have enabled surgeons to deepen their understanding of tumor localization and vascular anatomy before and during surgery, thus improving the safety and efficacy of surgery.

Finally, this study has limitations. First, although we included a certain number of patients for follow-up, a small number of patients were followed up for less than 3 years, and some patients did not observe endpoint events. Follow-up studies need to expand the number of cases further and extend the follow-up time to obtain more reliable conclusions. Second, this study used PSM to limit selection bias arising from insufficient randomization of the two groups of patients, but there may still be inherent biases not identified in retrospective studies.

## Conclusions

Our preliminary study showed that ICG fluorescence navigation laparoscopic hepatectomy for hepatocellular carcinoma is superior to conventional laparoscopic surgery in terms of long-term survival. This conclusion needs further large sample and multicenter data support.
